# NF-κB dependent and independent mechanisms of quartz-induced proinflammatory activation of lung epithelial cells

**DOI:** 10.1186/1743-8977-7-13

**Published:** 2010-05-21

**Authors:** Damien van Berlo, Ad M Knaapen, Frederik-Jan van Schooten, Roel PF Schins, Catrin Albrecht

**Affiliations:** 1 Institut für Umweltmedizinische Forschung (IUF) an der Heinrich-Heine-Universität Düsseldorf gGmbH, Germany; 2 Department of Health Risk Analysis and Toxicology, Maastricht University, the Netherlands; 3 Department of Toxicology and Drug Disposition, Schering-Plough, the Netherlands

## Abstract

In the initiation and progression of pulmonary inflammation, macrophages have classically been considered as a crucial cell type. However, evidence for the role of epithelial type II cells in pulmonary inflammation has been accumulating. In the current study, a combined *in vivo *and *in vitro *approach has been employed to investigate the mechanisms of quartz-induced proinflammatory activation of lung epithelial cells. *In vivo*, enhanced expression of the inflammation- and oxidative stress-related genes HO-1 and iNOS was found on the mRNA level in rat lungs after instillation with DQ12 respirable quartz. Activation of the classical NF-κB pathway in macrophages and type II pneumocytes was indicated by enhanced immunostaining of phospho-IκBα in these specific lung cell types. *In vitro*, the direct, particle-mediated effect on proinflammatory signalling in a rat lung epithelial (RLE) cell line was compared to the indirect, macrophage product-mediated effect. Treatment with quartz particles induced HO-1 and COX-2 mRNA expression in RLE cells in an NF-κB independent manner. Supernatant from quartz-treated macrophages rapidly activated the NF-κB signalling pathway in RLE cells and markedly induced iNOS mRNA expression up to 2000-fold compared to non-treated control cells. Neutralisation of TNFα and IL-1β in macrophage supernatant did not reduce its ability to elicit NF-κB activation of RLE cells. In addition the effect was not modified by depletion or supplementation of intracellular glutathione.

The results from the current work suggest that although both oxidative stress and NF-κB are likely involved in the inflammatory effects of toxic respirable particles, these phenomena can operate independently on the cellular level. This might have consequences for *in vitro *particle hazard testing, since by focusing on NF-κB signalling one might neglect alternative inflammatory pathways.

## Background

The highly abundant mineral quartz is present in nearly all rocks and minerals to some extent. Quartz can become fractured into very small (respirable) particles in various occupational settings, examples of which are mining, rock drilling, sandblasting, and highway construction. Worldwide, millions of workers are exposed to respirable crystalline silica; it is known for its ability to wreak havoc in the lung at high exposures and is associated with various pathologic conditions. In 1997, the International Agency for Research on Cancer (IARC) upgraded its evaluation of respirable quartz and classified it as a Group 1 human carcinogen [1], which was supported by a later cohort study in nearly 66.000 workers [2]. Quartz exposure can cause silicosis, a severely debilitating, often fatal disease which is characterized by chronic inflammation and persistent fibrosis. Disease progression is irreversible; the situation can deteriorate even when exposure is ceased. Currently, there is no effective treatment for silicosis. Other diseases associated with quartz exposure are COPD, tuberculosis, renal disease and autoimmune disease. A large body of evidence has been established supporting the role of the inflammogenic properties of quartz in the onset of quartz-induced disease. Exposure to high concentrations of respirable crystalline silica is well known to cause progressive fibrosis and lung cancer in rats. In humans, silicosis is associated with an increased risk of lung cancer. Based on chronic inhalation studies in the rat and epidemiologic evidence, the ability of quartz to elicit marked and persistent inflammation is believed to be a crucial factor for the development of these severe pathologies [3-5].

The transcription factor Nuclear Factor-kappa B (NF-κB) is pivotal in mediating inflammatory processes in general and is considered as the central regulator activating cells in response to silica [6]. In fact, NF-κB is also considered important for driving pulmonary toxicity of inflammogenic particles in general [7]. In its dormant state, NF-κB, which typically exists as a dimer of the RelA and p50 subunits, resides in the cytosol. Due to its binding to its inhibitor protein IκB, most commonly IκBα, it is unable to translocate into the nucleus. In the classical NF-κB activation pathway, the inhibitor protein IκBα is phosphorylated at serines 32 and 36 by the IKK enzyme complex, ubiquitinated at lysines 21 and 22, and subsequently degraded by the 26S proteasome. This unmasks the nuclear localization signal of NF-κB and liberates it from the nuclear export signal of IκBα, allowing NF-κB to migrate into the nucleus, where it binds to the DNA, and activates the transcription of many pro-inflammatory genes [7]. The acute inflammatory cytokines Interleukin-1 beta (IL-1β) and Tumor Necrosis Factor alpha (TNFα) are prime examples and potent inducers of NF-κB themselves [8-11], and are known to be involved in silicosis [12-14]. In addition, reactive oxygen and nitrogen species (ROS and RNS), which are generated in response to silica exposure and have been implicated in its adverse effects [14-17], are known to be capable of activating NF-κB [18,19]. Activation of NF-κB by silica has been shown *in vivo *[20-22], with activation localized to alveolar macrophages and pulmonary epithelial cells in particular [21,22].

In earlier particle toxicology research, the main cell type orchestrating the initial inflammatory response to inhaled particles was thought to be the alveolar macrophage. In the last decades however, evidence for a critical role of the alveolar epithelial type II cell has been building up. Crucially, the Blackwell group showed that *in vivo *expression of a dominant NF-κB inhibitor in mouse airway epithelium completely prevented lung inflammation and injury induced by lipopolysaccharide or a constitutively active form of IKKβ, an enzyme responsible for phosphorylation of the inhibitor of NF-κB (IκB) [23]. These findings show that NF-κB-regulated proinflammatory activation of airway epithelium is essential for the development of pulmonary inflammation. Although macrophages and alveolar lung epithelial cells are two main airway cell types likely to interact with inhaled particles, the contribution of each and the mechanisms involved in their proinflammatory activation are at present not fully known.

The aim of the current study was to investigate the mechanism of proinflammatory activation of lung epithelial cells by quartz. To achieve this, we investigated whether classical NF-κB pathway activation as well as expression of proinflammatory and oxidative stress-induced genes occurs *in vivo *in our rat model of quartz exposure. In parallel, we studied the mechanism of NF-κB activation by quartz particles (Dörentrup Quartz, DQ12) in RLE-6TN rat lung epithelial cells. The direct effect of quartz on RLE cells was compared to its indirect, macrophage product-mediated effect, assessed by treating RLE cells with (particle-free) supernatants of quartz-treated NR8383 rat alveolar macrophages.

## Methods

### Reagents

L-glutamine, Ham's F12 medium, Hanks' balanced salt solution with calcium and magnesium (HBSS^(+/+)^), fetal calf serum (FCS), penicillin/streptavidin solution, glutamine, phosphate buffered saline (PBS), bicinchoninic acid solution, copper(II)sulphate, Horseradish peroxidase (HRP), 5,5-dimethyl-1-pyrroline-N-oxide (DMPO), anti-mouse-IgG whole protein HRP-conjugated secondary antibody, tubulin antibody, hydrogen peroxide (H2O2), buthionine sulfoximine (BSO), N-acetyl cysteine (NAC) and diaminobenzidine were all obtained from Sigma (Germany). F12-K Nutrient Mixture and Trizol were obtained from Invitrogen (Germany). Protease inhibitors were purchased from Roche Diagnostics GmbH (Germany) as a Complete™ cocktail. ECL-reagent/detection system was obtained from GE Healthcare (Germany). Polyclonal antibodies against NF-κB RelA (sc-109) and IκBα (C-21, sc-371) were supplied by Santa Cruz Biotechnology (USA), antibody against phospho-IκBα (Ser32/36, monoclonal, clone 16A6) was purchased from Cell Signaling Technologies (USA). Recombinant rat IL-1β and TNFα, anti-IL-1β neutralizing antibody, anti-TNFα neutralizing antibody and ELISA kits for TNFα and IL-1β were purchased from R&D Systems (Germany). For immunohistochemical detection secondary biotinylated horse-anti mouse antibody, the streptavidin-biotin-system (Vectastain Elite Kit) and mouse IgG were purchased from Vector Laboratories (USA). Hoechst 33342 was bought from Sigma (Germany) and MFP555 goat anti-mouse IgG from MoBiTec (Germany). RNeasy^® ^mini kit and RNase-free DNase set were provided by Qiagen (Germany). The iScript cDNA Synthesis kit and SYBR© Green Supermix were bought from Biorad (Germany). NF-κB RelA binding ELISAs and nuclear extraction kits were obtained from Active Motif (Belgium). Isoflurane was bought from Essex Pharma (Germany). All other chemicals were of the highest purity and were supplied by Merck (Germany). RT-PCR primers were designed using Primer Express software and ordered from Operon (the Netherlands).

### Particles

Dörentrup quartz (DQ12 batch 6, IUF, Düsseldorf) with a mean diameter of 0.96 μm was used as a standard respirable quartz sample to expose animals and treat cell lines. In order to eliminate potential endotoxin contamination, quartz was baked at 220°C for 24 h. Immediately before intratracheal (i.t.) instillation of rats, quartz was suspended in phosphate-buffered saline (PBS) at a concentration of 5 mg/ml and sonicated for 5 min. To treat RLE and NR8383 cells, quartz was suspended in reduced RLE medium (Ham's F12 containing 0.1% FCS, 1% γ-glutamine, 1% penicillin and 1% streptomycin), respectively HBSS^(+/+) ^(EPR experiments) or normal NR8383 medium (other experiments, F12-K containing 15% FCS, 1% γ-glutamine, 1% penicillin and 1% streptomycin) at a concentration of 1 mg/ml and was sonicated for 5 min immediately prior to treatment.

### Animals and exposure

For the in vivo evaluation of NF-κB activation and mRNA expression, lung tissue and sections were used from an animal study which was previously performed in our lab [21,24]. Specific pathogen-free female Wistar rats (Janvier, Le Genest St. Isle, France) were housed and maintained in an accredited on-site testing facility, according to the guidelines of the Society for Laboratory Animals Science (GV-SOLAS). Food and water were available ad libitum. The animals were housed on hardwood bedding in plastic cages in an air-conditioned animal room (23 ± 2°C) with a regular 12 h light/dark cycle under SPF conditions. When they reached a weight of 200-250 g (8 weeks old), animals were anaesthetized with isoflurane and i.t. instilled with 2 mg DQ12 quartz (400 μl suspension) or the vehicle control only (400 μl PBS) using a laryngoscope. At 3 days after instillation, animals were deeply anesthetized with pentobarbital (50 mg/kg body weight) and sacrificed through exsanguination via the A. abdominalis.

### Culture and treatment of Rat Lung Macrophage Cell Line

NR8383 rat alveolar macrophage cells [25] were seeded in 60 mm culture dishes at a concentration of 0.5 × 10^6 ^per ml or, specifically for EPR measurements, cells were seeded in 24-wells plates at the same concentration. Cells were cultivated in Ham's F12K medium/15% fetal calf serum (FCS)/1% penicillin/1% streptomycin/1% glutamine for 3 days until they reached 60% confluence. Culture and treatments took place at 37°C and 5% CO_2_. Subsequently, cells were treated with 10, 40 or 80 μg/cm^2 ^quartz for 0.5 - 24 h as indicated for each specific experiment. Control cells received an equal volume of fresh NR8383 medium. After treatment, supernatants were harvested, either for direct measurement of ROS and cytokines using EPR and ELISA respectively, or for treatment of RLE cells. Obtained supernatants to be used for RLE treatment and ELISA were spun at low speed (200 g for 5 min) to remove non-adherent cells, and in order to remove cell debris and particles, they were spun once more at 18000 g for 5 min. Supernatants destined for EPR analysis were measured directly upon harvest.

### Culture and treatment of Rat Lung Epithelial Cell Line

Immortalized RLE cells [26] were seeded in 60 mm or 100 mm culture dishes at a concentration of 3 × 10^4 ^cells per cm^2 ^and cultivated in 2 or 6 ml (for 60 mm and 100 mm plates, respectively) Ham's F12 medium containing 5% FCS/1% penicillin/1% streptomycin/1% glutamine. At 90% confluence, medium was discarded and replaced by serum-reduced medium containing 0.1% FCS for 24 h for cell synchronization. Culture and treatments took place at 37°C and 5% CO2.

Cells were treated with quartz particles at concentrations of 10, 40 or 80 μg/cm^2 ^for 0.5-24 h as indicated. To compare the direct effects of quartz to the indirect (macrophage-mediated) effects, cells were additionally treated with 1:6 diluted (in serum-reduced RLE medium) supernatant of quartz-treated (10, 40 or 80 μg/cm^2^, 1-24 h) or non-treated control macrophages (Q-MS and c-MS, respectively). Based on pilot investigations, the highest dilution that still elicited a clear activation of the NF-κB pathway was selected. Control cells received fresh medium, for a period equal to the longest treatment. NF-κB and expression of the investigated genes were not induced by addition of fresh medium, at any time point up to 24 h (data not shown).

To investigate the potential of acute inflammatory cytokines and ROS to induce NF-κB activation in RLE cells, cells were treated with recombinant rat TNFα or IL-1β at concentrations of 0.1 up to 10 ng/ml as well as with hydrogen peroxide (H_2_O_2_) at a concentration of 50 μM for up to 2 hours. For the latter treatment, medium was taken off and was replaced by HBSS.

In order to inhibit cytokine activity, IL-1β and TNFα were inactivated using neutralizing antibodies. In a first step, effectiveness of inhibition was investigated by pre-treating recombinant TNFα and IL-1β diluted in macrophage medium for 1 h with neutralizing antibody at various concentrations. Cells were then treated with this mixture containing 1 ng/ml TNFα or 0.5-1 ng/ml IL-1β, the lowest concentrations found to induce a clearly visible degradation of IκBα as observed by Western blot. Optimal neutralizing antibody concentrations completely inhibiting cytokine-induced IκBα degradation were found to be 2.5 μg/ml for anti-IL-1β and 5 μg/ml for anti-TNFα.

Next, macrophage supernatants were pre-treated with either neutralizing antibody, or both simultaneously (2.5 μg/ml anti-IL-1β and 5 μg/ml anti-TNFα.), to inhibit cytokine activity. These conditioned media were then used to treat RLE cells.

To assess the influence of the intracellular redox status, intracellular glutathione was either depleted by incubating cells with 0.1 mM buthionine sulfoximine (BSO) for 24 h, or supplemented through treatment with 1 mM NAC for 2 h. BSO is a known inhibitor of glutathione synthesis, while NAC acts as a precursor for glutathione and provides the amino acid whose availability is rate-limiting for glutathione synthesis.

### Lung fixation and immunohistochemistry for phosphorylated (Ser32/36) IκBα

Lungs of 5 quartz-exposed and 5 control animals were instilled *in situ *with 4% paraformaldehyde/PBS (pH 7.4) under atmospheric pressure, removed, fixed in the same solution for at least 12 h, dehydrated, and paraffin embedded. After deparaffinization of 3 μm- lung sections with Xylene (2 × for 10 min), slides were washed successively for 10 min in acetone, acetone-TBS (1:1), and TBS. Subsequently, endogenous peroxidases were inactivated with 0.3% hydrogen peroxide for 30 min. After washing with TBS, sections were permeabilized in citrate buffer (pH 6.0) by microwave treatment and were washed again with TBS. Non-specific binding sites were blocked with normal horse serum (1:65) for 1 h, followed by incubation with a mouse monoclonal antibody against phospho-IκBα (Ser32/36). To allow for detection, slides were then incubated with a secondary biotinylated horse anti-mouse antibody (1:200) followed by the streptavidin-biotin-complex according to the manufacturer's protocol. Diaminobenzidine (DAB) was used as a substrate, and the slides were counter-stained with hematoxylin. As a negative control, serial sections were incubated with mouse IgG instead of the primary antibody at the same IgG concentration. Slides were analyzed using a light microscope (Olympus BX60).

### Quantitative RT-PCR analysis of gene expression in whole rat lung homogenate or RLE cells

Lungs of 3 quartz- and 3 saline-instilled animals as well as RLE cells were homogenized in Trizol. Subsequently RNA was extracted according to the manufacturer's instructions. The RNeasy^® ^mini kit coupled to DNAse treatment was used to purify total RNA from salts and residual DNA. Quantity and purity of RNA were evaluated using spectrophotometry at 230, 260, 280, and 320 nm. cDNA was synthesized using the iScript cDNA Synthesis kit, starting from 0.5 μg of RNA. cDNA was diluted 15× in water before use. PCR primers for rat HO-1, iNOS, COX-2 and the housekeeping gene HPRT were designed using Primer Express software (Applied Biosystems). Primer sequences for HO-1 were 5'-GGG AAG GCC TGG CTT TTTT-3' (forward) and 5'-CAC GAT AGA GCT GTT TGA ACT TGGT-3' (reverse), for iNOS 5'-AGG AGA GAG ATC CGG TTC ACA GT-3' (forward) and 5'-ACC TTC CGC ATT AGC ACA GAA-3' (reverse), for COX-2 5'-GCA CAA ATA TGA TGT TCG CAT TCT-3' (forward) and 5'-GAA CCC AGG TCC TCG CTT CT-3' (reverse), and for HPRT 5'-GCC CTT GAC TAT AAT GAG CAC TTC A-3' (forward) and 5'-TCT TTT AGG CTT TGT ACT TGG CTT TT-3' (reverse). qRT-PCR was performed with a MyiQ Single Color real time PCR detection system (BioRad) using SYBR© Green Supermix, 5 μl diluted cDNA, and 2.5 μl of 0.3 μM forward and reverse primer in a total volume of 25 μl. PCR was conducted as follows: a denaturation step at 95°C for 3 min was followed by 40 cycles at 95°C (15 seconds) and 60°C (45 seconds). After PCR, a melt curve (60-95°C) was generated for product identification and purity. PCR efficiency of all four primer sets, as assessed by the use of cDNA dilution curves, was 90-100%. Data were analyzed using the MyiQ Software system (BioRad) and were expressed as relative gene expression (fold increase) using the 2-ΔCt method [27].

### Immunocytochemistry for RelA in RLE cells

RLE cells were cultured to near confluence on 4-chamber culture slides (BD Falcon) for the immunocytochemical evaluation of the subcellular localization of RelA. After treatment, cells were washed in TBS, followed by incubation with 0.3% hydrogen peroxide for 30 min. After permeabilisation with 0.1% TritonX-100 for 5 min, non-specific binding sites were blocked with normal goat serum (1:65) for 1 h. For detection, cells were incubated with a rabbit polyclonal antibody against NF-κB RelA overnight at 4°C followed by a secondary goat anti-rabbit antibody (MFP555, 1:200). Cells were counter-stained with Hoechst 33342 (1 μg/ml). In order to control for unspecific staining, cells were incubated with rabbit IgG, instead of the primary antibody, at the same IgG concentration. Cells were analyzed using a fluorescence microscope (Olympus BX60) at an original magnification of ×1000.

### NF-κB RelA binding activity assay

To investigate DNA binding activity of NF-κB RelA in RLE nuclear extracts, an ELISA-based DNA binding activity assay [28] was performed.

Nuclear fractions of RLE cells were prepared using a nuclear extraction kit. To perform the assay, 5 μg of nuclear extract was transferred to each well and the assay was performed according to the manufacturer's recommendations. As a positive control, 2.5 μg Jurkat cell nuclear extract was used (supplied in the kit). Absorbance was read at 450 nm, while 650 nm was used as the reference wavelength.

### Western blotting

For Western blot analysis of IκBα and phospho-IκBα, whole cell extracts were prepared. RLE cells were harvested by gentle scraping and then lysed by 30 min incubation in 200 μl RIPA buffer (1% NP-40, 0.5% sodium deoxycholate, 0.1% sodium dodecyl sulfate in PBS) containing freshly added protease inhibitors at 4 C. Subsequently, they were centrifuged for 20 min at 15000 G and 4 C.

In the resulting supernatant, protein concentrations were determined using the bicinchoninic acid (BCA) assay, equal amounts (20 μg) were electrophoresed through 10% sodium dodecyl sulfate (SDS)-polyacrylamide gels, and transferred onto nitrocellulose membranes. Non-specific protein binding was blocked with 5% dried milk powder and 0.1% Tween-20 in PBS. Immunolocalization of IκBα protein was performed using a polyclonal antibody (1:1000) and an anti-rabbit-IgG whole protein HRP-conjugated secondary antibody (1:3000). To detect IκBα phosphorylated at serine 32 and/or 36, a monoclonal antibody was used (1:2000) in combination with an anti-mouse-IgG whole protein HRP-conjugated secondary antibody (1:2000). To normalize total protein amounts, blots were re-probed with an antibody against β-tubulin (1:5000) and a secondary anti-mouse-IgG whole protein HRP-conjugated antibody (1:5000). Band formation was visualized using an ECL Plus reagent/detection system. Quantification was performed by computer-assisted densitometry scanning using a documentation system with appropriate software (Gel-doc, Bio-Rad, Germany). Shown blots are representative for multiple experiments.

### ELISA

The presence of IL-1β and TNFα in macrophage media was quantified using ELISAs according to the manufacturer's recommendations (R&D Systems). TNFα and IL-1β concentrations were expressed in pg/ml and supernatant of non-treated cells was used as control.

### Electron paramagnetic resonance (EPR)

For the assessment of the quartz-induced generation of ·OH by alveolar macrophages, NR8383 cells were cultured in 24-wells plates for 3 days. Before quartz treatment, cells were incubated for 30 min in HBSS containing calcium and magnesium (HBSS^(+/+)^). NR8383 cells were treated with freshly sonicated quartz at a concentration of 10 or 40 μg/cm^2 ^for 1 up to 3 h at 37°C in the presence of 0.11 M 5,5-dimethyl-1-pyrroline-N-oxide (DMPO). Supernatant was transferred to a 100 μl glass capillary and ·OH generation was measured using a Miniscope MS200 EPR spectrometer (Magnetech, Berlin, Germany). EPR spectra were recorded at room temperature using the following instrumental conditions: Magnetic field: 3,360 G; sweep width: 100 G; scan time: 30 s; number of scans: 3; modulation amplitude: 1,800 G. The EPR signal, generated in the form of a DMPO-OH peak quartet was quantified; the amplitudes were averaged and expressed in arbitrary units (A.U.).

### Glutathione assay

To determine the efficiency of glutathione depletion or supplementation using BSO and NAC respectively, the intracellular total glutathione content was measured using the method developed by Tietze [29].

RLE cells were cultured in 6-well plates for 3 days until confluency was reached. At that point they were harvested by incubating cells with 500 μl trypsin-EDTA per well for 5 min at 37°C. After 5 min, 1 ml ice-cold culture medium was added. Next, cells were spun at 140 G for 5 min at 4°C and washed twice with 1 ml ice-cold PBS. Ice-cold extraction buffer (0.1% Triton/0.6% Sulfosalicylic acid in KPE buffer) was added to the cell pellet, which was subsequently homogenized using a Teflon pestle, and alternatingly vortexed and sonicated 3 times for 20 respectively 30 sec. Cell debris were collected by centrifugation at 3500 G for 5 min at 4°C and the supernatant was kept on ice until analysis.

Briefly, 20 μl of each sample was mixed with 5,5'-Dithiobis(2-nitrobenzoic acid) (DTNB) and glutathione reductase, and GSSG (reduced glutathione) was allowed to be converted to the oxidized form GSH for 30 sec, after which β-NADPH was added. The rate of change in absorbance was measured at 412 nm. The calculated glutathione concentration was corrected for the total protein concentration and expressed as % of control.

### Statistical evaluation

Data are expressed as mean ± SD, unless stated otherwise. Student's T-test was used to evaluate mRNA expression in the lungs of quartz versus saline exposed rats. A difference was considered to be statistically significant when p < 0.05. All *in vitro *experiments represent at least two independent experiments. NF-κB activation (as measured by ELISA), production of TNFα, IL-1β and ROS as well as mRNA expression changes were evaluated by ANOVA with Dunnett's posthoc comparison method. Effects are indicated at p < 0.05 and p < 0.01.

## Results

### NF-κB is activated in vivo in rats after quartz exposure

**Figure 1:**
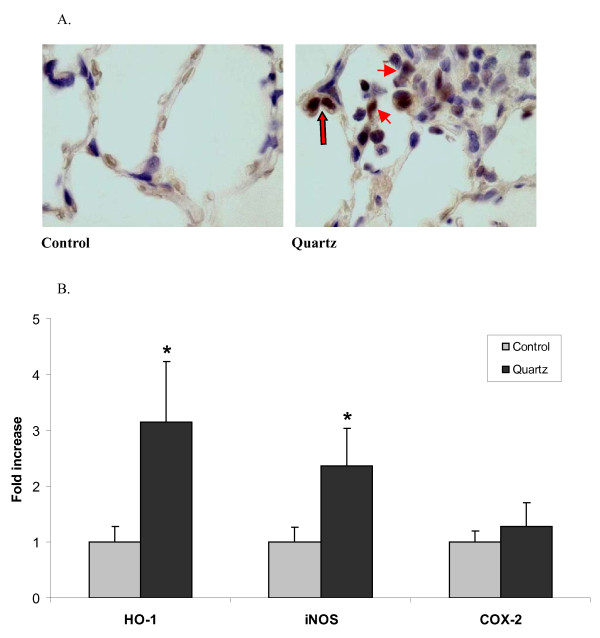
***In vivo *NF-κB activation and expression of inflammation-related genes in quartz-instilled rats, 3 days post-exposure**. **A**. Representative immunohistochemistry of lung tissue sections from rats instilled with DQ12 quartz or PBS. Dark brown staining indicates Ser32/36 phospho-IκBα, blue/purple staining indicates cell nuclei. Short arrows point out alveolar epithelial type II cells, alveolar macrophages are designated by the longer arrow. Original magnification: 1000×. **B**. *In vivo *expression of iNOS, HO-1 and COX-2 in lung tissue homogenates measured by qRT-PCR. Data are expressed as fold induction compared to control animals and are corrected for expression of the housekeeping gene HPRT. *P < 0.05 vs. control animals (n = 3/group).

To determine the effects of respirable quartz exposure on *in vivo *activation of the classical NF-κB pathway, lung tissue sections of quartz-exposed rats were analyzed for Ser32/36-phosphorylated IκBα by immunohistochemistry. Moreover, we investigated gene expression changes of the NF-κB-regulated genes iNOS and COX-2, as well as the oxidative stress marker heme oxygenase-1 (HO-1) in whole lung tissue homogenates obtained from quartz-instilled animals and control animals by qRT-PCR. Representative pictures of lung sections stained for Ser32/36-phosphorylated IκBα are shown in Fig. 1A. Marked immunoreactivity was observed in both alveolar macrophages and alveolar epithelial type II cells. In contrast, Ser32/36-phosphorylated IκBα was not observed in tissue sections of saline-instilled lungs. In the quartz-instilled group significantly higher mRNA expression of iNOS and HO-1 was found, as is shown in Fig. 1B. The mRNA expression of COX-2 was also slightly increased but this did not reach statistical significance (Fig. 1B).

### Mediators released from quartz-treated macrophages are stronger activators of NF-κB in rat lung epithelial cells than quartz particles

**Figure 2:**
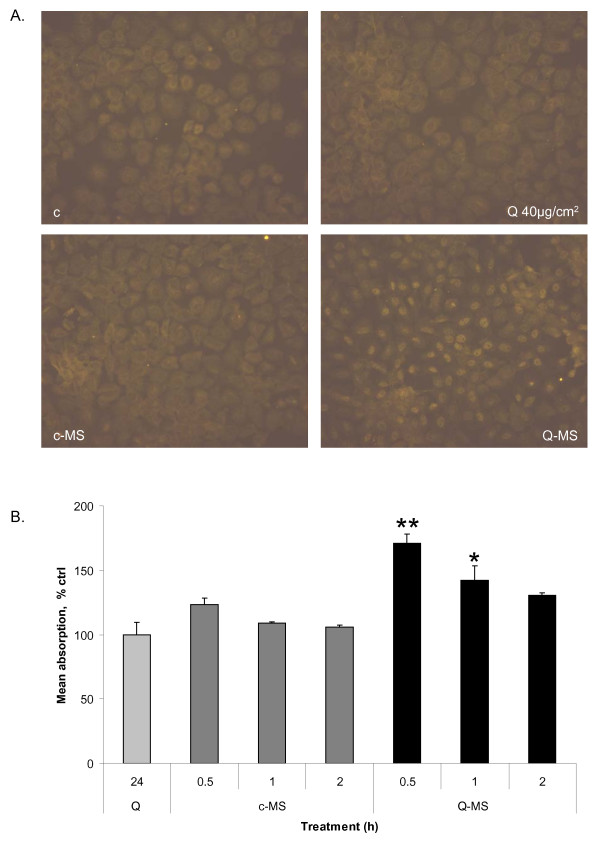
**Effects of quartz and supernatants from quartz-treated macrophages on epithelial NF-κB pathway activation A**. Immunocytochemistry of RelA nuclear translocation in RLE cells treated with supernatants from quartz-treated macrophages (*Q-MS*), untreated control macrophages (*c-MS*), or after direct treatment with quartz (*Q*). These are compared with untreated control cells (*c*). Cells were treated for 30 min with Q-MS and c-MS or quartz. Orange staining indicates the presence of the RelA subunit of the NF-κB complex. Original magnification: 400×. **B**. DNA binding activity of nuclear extracts from RLE cells treated with 40 μg/cm^2 ^quartz for 24 h (*Q*), or with supernatants from untreated (*c-MS*) or quartz-treated (*Q-MS*) macrophages for 30, 60 or 120 min. Data are expressed as percentage of non-treated controls. *P < 0.05 and **P < 0.01 vs. control (100%).

**Figure 3:**
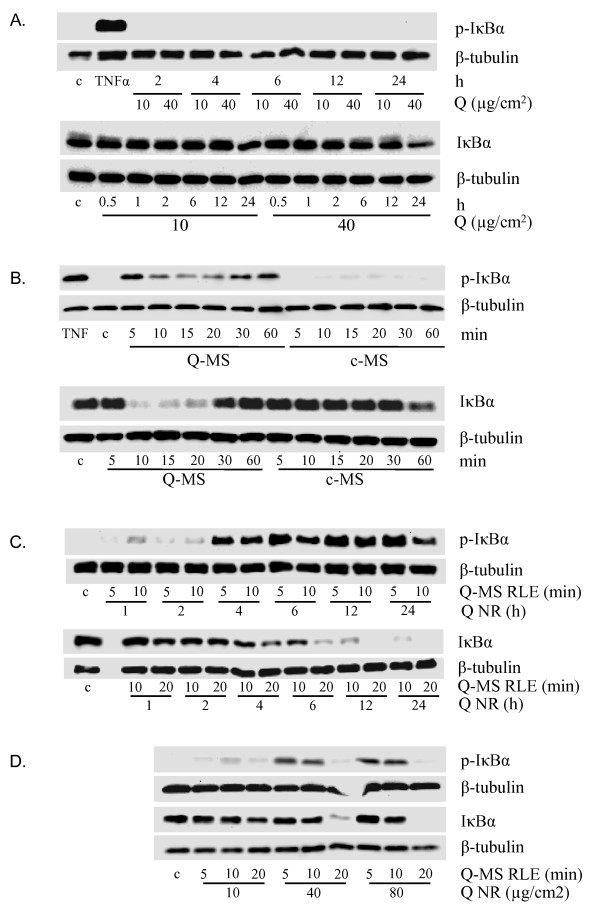
**NF-κB activation in RLE cells by quartz particles compared to indirect, macrophage mediator-induced NF-κB activation**. **A **Western blot analysis of Ser32/36 phosphorylation and IκBα degradation in whole cell extracts of RLE cells treated with 10 or 40 μg/cm^2 ^quartz (*Q*)for 0.5 to 24 h. TNFα (10 ng/ml, 10 min) was used as positive control for Ser32/36 phosphorylation (*c *= untreated cells). β-tubulin is used as loading control. **B**. Ser32/36-phosphorylation and IκBα degradation in RLE cells treated for 5 to 60 min with supernatants of quartz-treated (*Q-MS*) or untreated (*c-MS*) NR8383 cells (*c *= untreated cells). Macrophages were treated with 40 μg/cm^2 ^DQ12 or medium for 24 h. TNFα (10 ng/ml, 10 min) was used as positive control for Ser32/36 phosphorylation. β-tubulin served as loading control. **C**. IκBα phosphorylation and degradation in RLE cells treated for 5 to 20 min with supernatants harvested from macrophages that were previously treated with 40 μg/cm^2 ^quartz for 1 to 24 h (*c *= untreated cells; *Q-MS RLE *= treatment time for RLE cells with the macrophage supernatants; *Q NR *= treatment time for NR8383 macrophages with quartz). β-tubulin was used as loading control. **D**. IκBα phosphorylation and degradation in RLE cells treated with supernatants harvested from NR8383 macrophages treated for 24 h with 10, 40 or 80 μg/cm^2 ^quartz (*c *= untreated cells; *Q-MS RLE *= treatment time for RLE cells with the macrophage supernatants; *Q NR *= treatment concentration for NR8383 macrophages with quartz). As loading control, blots were reprobed with a β-tubulin-antibody.

To compare NF-κB activation induced by quartz particles (direct effect) or by mediators released from quartz-treated macrophages (indirect, macrophage-mediated effect) in RLE cells, NF-κB activation was determined on four levels: Nuclear translocation of the RelA subunit of NF-κB was visualised by immunocytochemical analysis, RelA NF-κB binding activity was measured using a binding activity assay and finally IκBα phosphorylation and degradation were investigated by Western blotting. The latter two endpoints are specific indicators of the classical NF-κB activation pathway. Supernatants from quartz-treated NR8383 macrophages (Q-MS) were found to elicit marked RelA nuclear translocation in RLE cells, whereas treatment with quartz and supernatants from untreated control macrophages (c-MS) did not (Fig. 2A). Q-MS also increased the DNA binding affinity of RelA in RLE nuclear extracts, in contrast to quartz treatment (Fig. 2B). Finally, Q-MS were shown to induce a fast and strong degradation of IκBα, preceded by phosphorylation (Fig. 3B). In contrast, direct quartz treatment induced only a mild reduction of the steady-state level of IκBα after a 24 h treatment with 40 μg/cm^2 ^quartz, in the absence of any detectable IκBα phosphorylation (Fig. 3A). Replacing medium only for up to 24 h did not result in detectable NF-κB activation (data not shown). The fast phosphorylation (5 min) and subsequent degradation of IκBα (10 min) induced by Q-MS was followed by a rapid restoration (30 to 60 min) due to its *de novo *synthesis (Fig. 3B).

By treating NR8383 macrophages with quartz for periods of 1 up to 24 h, we found that supernatants obtained from cells treated for at least 4 h with quartz were able to activate NF-κB in RLE cells, as observed by IκBα phosphorylation and degradation (Fig. 3C). The strongest effects are observed with Q-MS from macrophages treated for 12 or 24 h. Additionally, we observed that the NF-κB activating properties can only be attributed to conditioned media obtained from macrophages that were treated with high quartz concentrations (i.e. 40 or 80 μg/cm^2^) (Fig. 3D). When taken together, our data suggest that in quartz exposed lungs, NF-κB activation in lung epithelium occurs predominantly via indirect mechanisms, mediated by macrophage products, rather than directly through the quartz particles themselves.

### Macrophages produce ROS and acute inflammatory cytokines in response to quartz in a time- and dose-dependent manner

**Figure 4:**
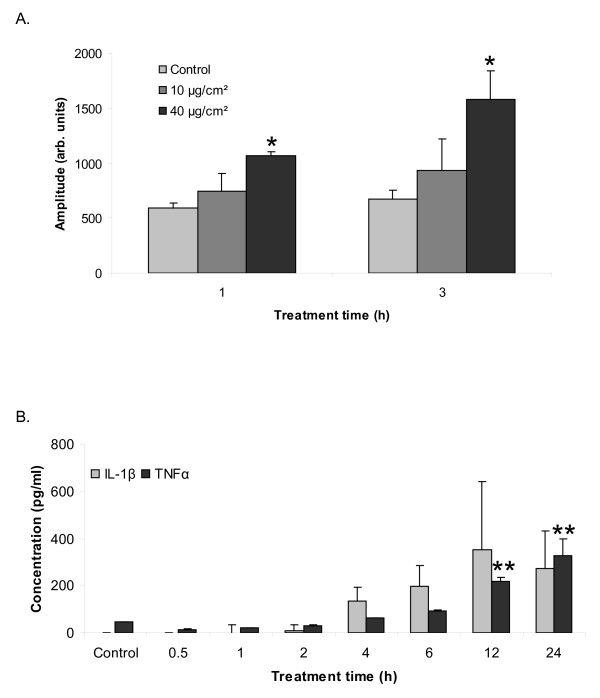
**Release of ROS, IL-1β and TNFα by quartz-treated NR8383 macrophages**. **A**. Reactive oxygen species (ROS) formation by NR8383 macrophages in response to quartz treatment for 1 and 3 h at concentrations of 10 and 40 μg/cm^2^, measured by EPR spectroscopy in combination with the spin trap DMPO. The mean amplitude of the four peaks representing DMPO-OH adducts is expressed in arbitrary units. **B**. IL-1β and TNFα concentrations (pg/ml) measured by ELISA in non-diluted supernatants of NR8383 macrophages which were treated with 40 μg/cm^2 ^quartz for 0.5 to 24 hours. *P < 0.05 and **P < 0.01 vs. control.

The ability of quartz to elicit ROS production from NR8383 cells was determined by EPR coupled to DMPO spin trapping. Quartz was found to induce ROS generation from the NR8383 cells in both a dose- and time-dependent manner (Fig. 4A). To investigate the role of the inflammatory cytokines IL-1β and TNFα in macrophage-mediated NF-κB pathway activation in the RLE cells, their presence in Q-MS was measured by ELISA. Treatment of NR8383 cells with 40 μg/cm^2 ^quartz elicited a marked release of TNFα and IL-1β (Fig. 4B), reaching a maximum value of around 0.3 ng/ml for both cytokines after 12-24 h of treatment.

### Neutralizing antibodies are capable of fully blocking TNFα- and IL-1β-induced NF-κB activation, but do not reduce Q-MS -induced activation of NF-κB

**Figure 5:**
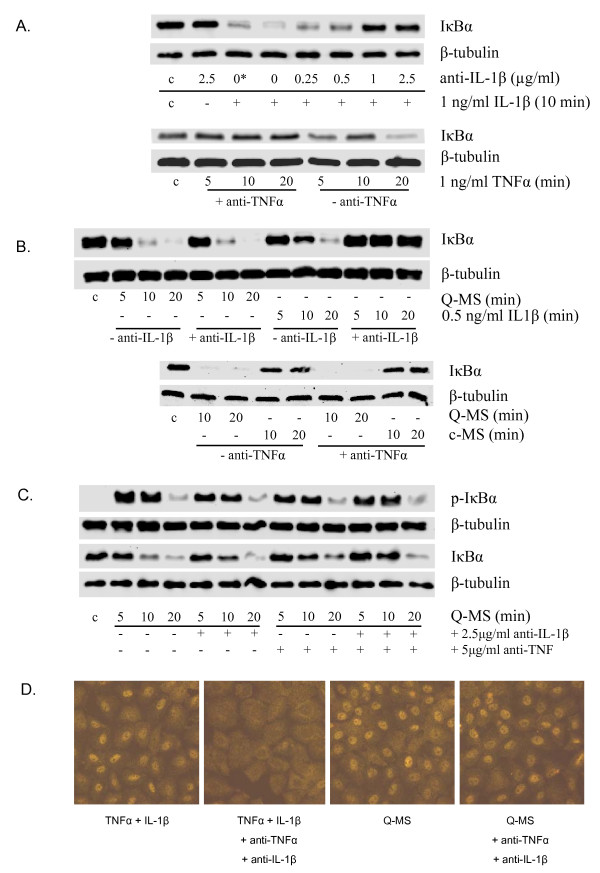
**Role of IL-1β and TNFα in macrophage-mediated NF-κB activation in RLE cells**. **A**. Western blot analysis of IκBα degradation in RLE cells treated with 1 ng/ml IL-1β for 10 min or with 1 ng/ml TNFα for 5, 10 and 20 min, both pre-treated with neutralising antibodies. For neutralisation, the cytokine solution was incubated for 1 h with 0.25-2.5 μg/ml anti-IL-1β or 5 μg/ml anti-TNFα antibody. The asterisk indicates cells treated with IL-1β incubated at 37°C for 1 h, to investigate incubation effects. β-tubulin antibody was used as loading control. **B**. Expression of IκBα protein in RLE cells after treatment with supernatants of quartz-treated (40 μg/cm^2^, 24 h) NR8383 cells (*Q-MS*), that were pre-treated with neutralising antibodies (5 μg/ml anti-TNFα or 2.5 μg/ml anti-IL-1β). Supernatant of non-treated (medium, 24 h) macrophages (*c-MS*) was used as negative control for the TNFα neutralisation, β-tubulin served as loading control. **C. and D**. Effect of pre-treatment with both neutralising antibodies on NF-κB activation determined by Western blot (**C**) (phospho-IκBα, total IκBα) and (**D**) Immunocytochemistry (RelA). For Western blot experiments, RLE cells were treated for 5 to 20 min with native or cocktail-treated Q-MS. For immunocytochemical analysis, cells were treated with Q-MS or a cytokine mix (0.125 ng/ml IL-1β + 0.25 ng/ml TNFα) for 15 min. Macrophages were treated with 40 μg/cm^2 ^DQ12 or medium for 24 h. Original magnification: 1000×. Pre-treatment conditions for both methods consisted of 1 h incubation with both 5 μg/ml anti-TNFα and 2.5 μg/ml anti-IL1β. β-tubulin was used as loading control.

In Fig.5A, the ability of anti-TNFα and anti-IL-1β neutralizing antibodies to block activation of NF-κB in RLE cells by their respective cytokine targets is demonstrated by Western blot analysis of IκBα. The same concentration of neutralizing antibodies shown to abrogate TNFα- and IL-1β-induced NF-κB activation in RLE cells was then used to pre-treat Q-MS, before addition to RLE cells. Q-MS contained markedly less TNFα and IL-1β than was used for the treatments with each respective cytokine, therefore the neutralising antibody concentrations should be more than sufficient. No visible decrease in the NF-κB-activating potential could be observed after pretreatment with either antibody (Fig. 5B). To account for compensatory effects, a mixture of both neutralizing antibodies was also used as Q-MS pre-treatment. However, even blocking the activity of both cytokines did not result in an inhibition of IκBα Ser32/36 phosphorylation and IκBα degradation or RelA nuclear translocation (Fig. 5C). These results suggest that neither TNFα nor IL-1β do play an essential role in NF-κB activation in lung epithelial cells induced by macrophage products.

### Modulation of the intracellular glutathione level does not affect Q-MS-induced activation of NF-κB

**Figure 6:**
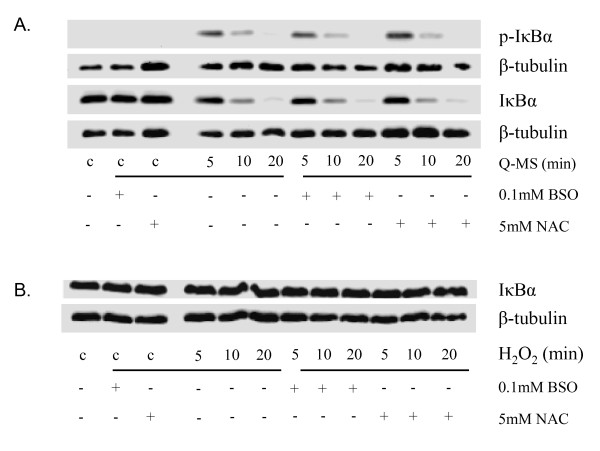
**Role of ROS and cellular glutathione status in macrophage-mediated NF-κB activation in RLE cells**. **A**. Modulation of intracellular glutathione levels to investigate the influence of intracellular redox status on phosphorylation and degradation of IκBα induced by supernatant of quartz-treated macrophages (*Q-MS*). Macrophages were treated with 40 μg/cm^2 ^DQ12 or PBS for 24 h. RLE cells were pretreated with 0.1 mM buthionine sulfoximine (BSO) for 24 h to deplete glutathione or with 1 mM N-acetyl cysteine (NAC) for 2 h to enhance intracellular levels. IκBα and phospho-IκBα protein was visualized using Western blot; blots have been reprobed for β-tubulin as a loading control. **B**. Investigation of the direct potential of ROS to activate the classical NF-κB pathway by measuring IκBα degradation using Western blotting. RLE cells were treated with 50 μM hydrogen peroxide (H_2_O_2_) in a medium-free environment for 5-20 min. Additionally, the intracellular glutathione level was modulated using BSO and NAC at concentrations of 0.1 mM and 1 mM. β-tubulin served as loading control.

To study the influence of the intracellular redox-status on Q-MS-induced NF-κB activation, glutathione was depleted in RLE cells using buthionine sulfoximine (BSO), or supplemented by NAC treatment. Treatment of RLE cells with BSO was found to cause a dose-dependent depletion of intracellular glutathione to a minimum of 5% of the control level whereas treatment with the glutathione precursor NAC was found to enhance intracellular glutathione levels up to a maximum of 133% (data not shown). Concentrations inducing a maximal effect (0.1 mM BSO and 1 mM NAC) were used in subsequent experiments. These experiments showed that, irrespective of the intracellular glutathione status, Q-MS treatment caused IκBα phosphorylation and its subsequent degradation (Fig. 6A). These observations indicate that the intracellular antioxidant status does not modulate NF-κB activation by macrophage mediators in our model.

### ROS do not activate NF-κB in RLE cells

To investigate the potential role of ROS in Q-MS-induced NF-κB activation in RLE cells, cells were pre-treated with BSO and NAC as described before. Then, they were treated with 50 μM H_2_O_2 _in HBSS. H_2_O_2 _failed to activate NF-κB in the RLE cells, as there was no visible IκBα Ser32/36 phosphorylation, IκBα degradation (Fig. 6B) or increased RelA-DNA-binding activity (data not shown).

### Contrasting induction of iNOS, COX-2 and HO-1 mRNA by Q-MS and quartz

**Figure 7:**
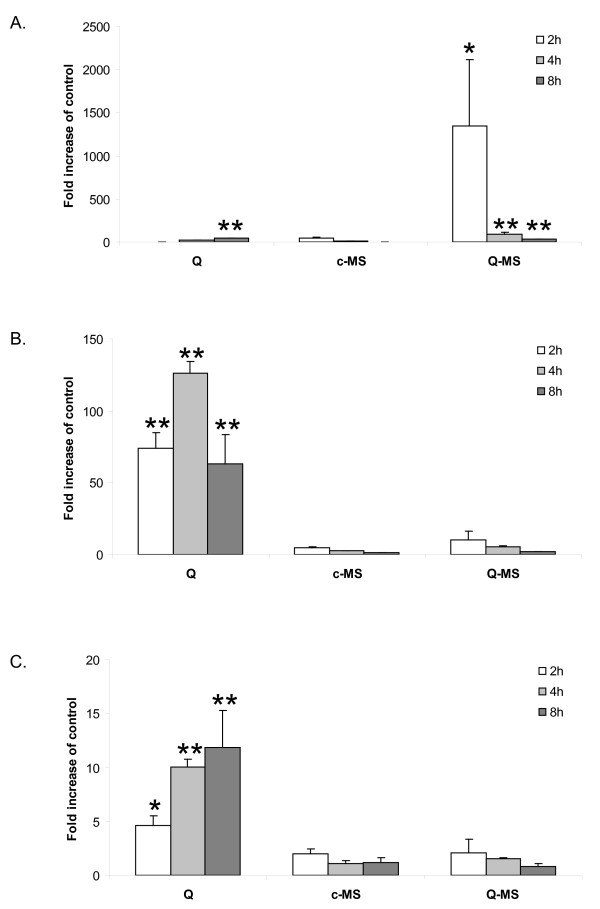
**Direct and indirect quartz effects on iNOS, COX-2 and HO-1 mRNA expression in RLE cells**. Quantitative RT-PCR determination of mRNA levels of iNOS (**A**), COX-2 (**B**) and HO-1 (**C**) in RLE cells after treatment with quartz (*Q*) and supernatants of quartz-treated macrophages (*Q-MS*) for 2, 4 or 8 h. As an extra control, cells were additionally treated with supernatants of non-treated macrophages (*c-MS*). Data are expressed as fold increase of mRNA levels in non-treated control cells and were corrected for the housekeeping gene β-actin. *P < 0.05 and **P < 0.01 vs. control.

To investigate and compare the effect of quartz and Q-MS on NF-κB activation in RLE cells at the mRNA level, cells were treated with quartz and Q-MS for 2, 4 and 8 h, subsequently gene transcripts of the NF-κB-regulated genes *inos *and *cox-2 *were measured using qRT-PCR analysis. c-MS served as negative control. Q-MS was capable of strongly and rapidly inducing iNOS, as shown by an over 1300-fold increase after 2 h, that faded quickly at later time points. Direct treatment of the epithelial cells with quartz elicited a gradual increase, reaching a maximum of 40-fold after 8 h (Fig. 7A). COX-2 was induced at all time points by quartz up to 125-fold after 4 h, while Q-MS was a fast but relatively weak inducer of COX-2, peaking at a 10-fold increase after 2 h (Fig. 7B).

To investigate the observed effects in relation to oxidative stress, transcript levels of HO-1 were measured using the same method. Quartz induced a time-dependent increase of up to 12-fold, while no indirect quartz effect was found in the supernatant-treated cells (Fig. 7C). The qRT-PCR data obtained in this study indicate that iNOS, which was shown to be upregulated *in vivo *in the rat lung after quartz exposure, can be highly induced in rat lung epithelial cells by products of quartz-treated macrophages, while to a lesser degree and via more gradual kinetics by quartz. In sharp contrast to these findings, COX-2 mRNA was mainly increased by direct quartz treatment. Analysis of HO-1 mRNA demonstrated that only quartz and not Q-MS induces oxidative stress in rat lung epithelial cells.

### Pre-coating quartz with PVNO fully inhibits proinflammatory effects of Q-MS and quartz particles

**Figure 8:**
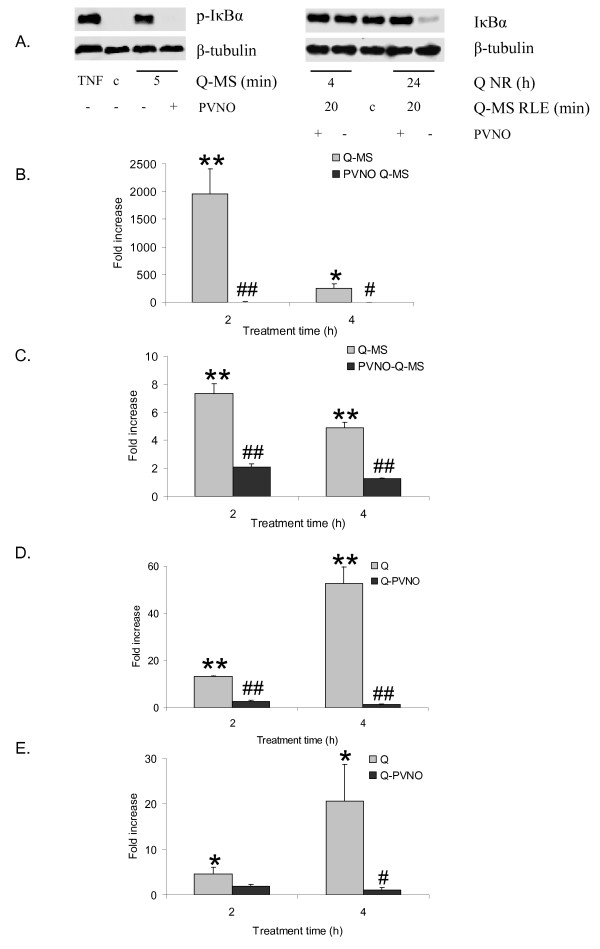
**Effects of quartz particle surface modification on NF-κB activation and mRNA expression in RLE cells**. **A**. Western blot analysis of Ser32/36 phospho-IκBα and total IκBα expression in RLE cells treated for 20 min with supernatants of macrophages (*Q-MS RLE*) that were treated either with native quartz or PVNO-modified quartz for 24 h (*Q-NR*). For phospho-IκBα analysis, TNFα (10 ng/ml, 10 min) was used as positive control. As loading control, blots were reprobed with an antibody against β-tubulin. **B and C**. Comparison of mRNA levels of iNOS (**B**), and COX-2 (**C**) in RLE cells treated with supernatant of macrophages treated with native quartz (*Q-MS*) or macrophages treated with PVNO-coated quartz (*PVNO Q-MS*) for 2 h and 4 h, measured using quantitative RT-PCR. Data are expressed as the fold increase of control mRNA levels and were corrected for the housekeeping gene β-actin. **D and E**. Comparison of mRNA levels of COX-2 (**D**), and HO-1 (**E**) in RLE cells treated with 40 μg/cm^2 ^quartz (*Q*) or 40 μg/cm^2 ^PVNO-coated quartz (*Q-PVNO*) for 2 or 4 h, measured using quantitative RT-PCR determination. Data are expressed as the fold increase of control mRNA levels and were corrected for the housekeeping gene β-actin. *P < 0.05 and **P < 0.01 vs. control, ^# ^*P *< 0.05 and ^## ^*P *< 0.01 vs. Q-MS (panel A, B) or vs. Q (panel C, D).

As inhibition at the macrophage product level proved to be ineffective, an intervention upstream of the pro-inflammatory activation cascade was performed by modifying the quartz surface with polyvinyl pyridine N-oxide (PVNO). Treatment of RLE cells with supernatant of macrophages treated for 24 h with 40 μg/cm^2 ^DQ12-H_2_O, a non-coated quartz sample prepared in the same way as PVNO-coated quartz, resulted in a clear IκBα phosphorylation and degradation after 5 - 20 min (Fig. 8A). Also, the iNOS transcription was strongly induced after 2 h as seen before using standard Q-MS. When cells were treated with PVNO-Q-MS however, IκBα phosphorylation and degradation were not visible and induction of iNOS mRNA was nearly abrogated (Fig. 8B). Also, induction of COX-2 expression by Q-MS was all but eliminated by PVNO coating (Fig. 8). Investigation of the direct particle effect of PVNO-quartz on COX-2 and HO-1 expression showed a similar trend: induction of the transcription of both genes was powerfully inhibited (Fig. 8D, E). These combined findings suggest that PVNO coating of quartz provides effective protection against: (1) macrophage mediated, NF-κB-dependent activation, as well as (2) direct, NF-κB-independent, activation of inflammatory mediators in lung epithelium.

## Discussion

The present study was undertaken to address the mechanisms of NF-κB activation in lung epithelium by respirable quartz particles. In a previous study, we could already show that quartz exposure leads to an increased nuclear staining of the NF-κB subunit RelA in type II epithelial cells as well as alveolar macrophages [21]. Now, we have provided further *in vivo *evidence for the actual activation of the classical NF-κB pathway in both cell types, the pathway involving phosphorylation of IκBα at serines 32/36, tagging this inhibitor protein for degradation. In our *in vivo *model, the observed activation of NF-κB in rat lung tissue was accompanied by increased mRNA expression of iNOS, which has been shown to be involved in inflammation [14,17] and to be induced on the mRNA level by silica exposure in rat lung [30]. iNOS is known to be regulated at least partly by NF-κB [31-34]. A classic pro-inflammatory gene induced by NF-κB is COX-2, known to occupy an important position in the regulation of pulmonary inflammation [35]. Although the role of COX-2 in inflammation is complex and the concept of a general pro-inflammatory role has been disproved [36], in the acute stage of pulmonary disease COX-2 expression is considered to promote inflammation [35]. COX-2 is known to be mainly regulated on the level of transcription [35] and the gene has been shown to be induced in lung epithelial type II cells [37]. In the current study, COX-2 was not significantly induced *in vivo *following quartz exposure on the mRNA level. In contrast, we observed a significant increase of HO-1 mRNA expression in quartz exposed rat lungs. HO-1 induction is considered as a sensitive oxidative stress marker [38] and has been shown to be increased in serum of silicotic patients as well as in lung tissue of silica-exposed mice [39,40]. Our findings provide further support for the well-known role of oxidative stress in silica-elicited pathogenesis [15,16].

Our parallel *in vitro *approach focused on the investigation of the role of macrophages in NF-κB activation in lung epithelial cells. Therefore, we compared epithelial NF-κB activation in response to treatment with cell-free supernatants from quartz-treated macrophages to the direct treatment with quartz particles. Evaluation of NF-κB pathway activation on multiple levels is necessary, as there might be different mechanisms involved. While cytokines like TNFα and IL-1β induce the canonical pathway characterized by IκBα Ser32/36-phosphorylation and degradation, quartz particles have also been shown to activate NF-κB in macrophages via tyrosine phosphorylation of IκBα [41]. In the present study, we observed that supernatants of quartz-treated NR8383 alveolar macrophages caused Ser32/36 phosphorylation and degradation of IκBα, the main NF-κB inhibitor protein, in epithelial cells. These supernatants were also found to cause nuclear translocation and DNA binding of the RelA NF-κB subunit, part of the most common RelA-p50 heterodimer that is generally known as NF-κB. Only RelA contains a transactivation domain and thus induces transcription of inflammatory genes [42], while p50 homodimers are known to inhibit transcription by competing for and blocking available DNA docking sites [43]. In association with NF-κB pathway activation, supernatants from the quartz-treated macrophages also caused a massive increase in the number of iNOS gene transcripts in the RLE rat lung epithelial cells. In sharp contrast to these findings, direct treatment with quartz only induced a marginal reduction of IκBα levels after the longest treatment. Apart from reflecting active degradation, the observed IκBα decline may also be a result of an inhibited *de novo *synthesis [44]. In association with this, quartz itself was also found to be a rather weak inducer of iNOS, when compared to supernatants of the quartz-treated macrophages. However, direct treatment with quartz resulted in a more than 100-fold upregulation of COX-2 mRNA in the RLE cells, an effect that was much stronger than that observed with the macrophage supernatants. An NF-κB-independent induction of COX-2 by silica appears to contrast with previous findings by Choi and colleagues [45]. However, in their study fibroblasts were used, and cell type-specific differences in COX-2 regulation are a possibility. Although COX-2 expression is considered to be regulated through NF-κB signalling, other transcription factors have been implicated as well, both in general [46,47] and specifically after silica treatment [48]. For instance, in lung epithelial cells, it has been shown that zinc-induced COX-2 induction is not reduced by NF-κB inhibition [49].

To further investigate the mechanism of NF-κB activation in the lung epithelial cells, we evaluated the roles of the most likely relevant candidates, i.e. the pro-inflammatory cytokines TNFα and IL-1β, as well as the involvement of ROS. TNFα and IL-1β are considered important factors in the development of silicosis [12-14] and are well-known inducers of NF-κB in various cell types [8-11]. Both cytokines were produced by the NR8383 cells upon quartz treatment, and the concentrations as measured in the supernatants of macrophage treated with quartz for various periods matched well with the ability of these supernatants to activate NF-κB. Remarkably however, inhibition of TNFα or IL-1β with their respective neutralizing antibodies did not influence NF-κB-inducing potential of the supernatants from quartz-treated NR8383 cells. Even when both cytokines were neutralized simultaneously in the supernatants from the quartz-treated macrophages, epithelial NF-κB activation could not be inhibited. Taken together, these findings suggest that neither TNFα nor IL-1β plays a crucial role in quartz-induced NF-κB activation in lung epithelial cells, and that other factors are at least involved or possibly even more important. This is in accordance with previous investigations in our laboratory, which indicated that *in vivo *NF-κB activation by quartz may be, at least in part, TNFα-independent [21].

To address the potential role of ROS in the macrophage-mediated NF-κB activation in RLE cells the effects of H_2_O_2_, BSO and NAC were evaluated. An H_2_O_2 _concentration of 50 μM was used, as it was shown to induce a strong increase in NF-κB DNA binding activity in Jurkat cells [19], while in addition, in our lab it proved to be one of the highest concentrations applicable without causing significant cytotoxicity in RLE cells (data not shown). H_2_O_2 _is well known to possess NF-κB activating properties, albeit in a cell specific-manner [19,50]. H_2_O_2 _is formed upon the dismutation of superoxide anions which are generated in large amounts by activated macrophages during inflammation. Although the generation of ROS from DQ12-treated NR8383 macrophages was shown in this study in a concentration and time dependent manner, H_2_O_2 _treatment did not lead to any notable IκBα degradation in the RLE cells. This observation might be due to the fact that H_2_O_2 _has been found to activate NF-κB through a non-classical pathway in some cell types, involving tyrosine phosphorylation of IκBα and direct activation of RelA [51]. However, nuclear extracts from H_2_O_2 _treated RLE cells failed to increase DNA binding activity of RelA in our hands (data not shown). In line with these observations, we also did not observe any effect of the macrophage supernatants on NF-κB pathway activation in RLE cells upon modification of the intracellular glutathione content with BSO or NAC. In alveolar epithelial cells, glutathione is considered to be the most important intracellular antioxidant [52]. Lower levels of intracellular glutathione have been associated with enhanced NF-κB activation [53]. In Jurkat cells, increasing intracellular glutathione levels using NAC has been shown to block H_2_O_2_-induced NF-kB activation [19]. There is also evidence for the interplay between ROS and cytokines in NF-κB induction [18]. In our hands however, depleting the intracellular glutathione concentration to 5% of its normal level using BSO or augmenting it to 133% using NAC did not influence NF-κB activation in macrophage supernatant-treated or H_2_O_2_-treated RLE cells. It remains to be investigated whether in our experiments the protective role of glutathione is taken over by other intracellular thiols or thioredoxin [54].

Our findings suggest that oxidative stress is not a key mechanism in the activation of NF-κB in lung epithelial cells by quartz. This is evidenced by the inability of H_2_O_2 _to directly activate NF-κB, as well as the finding that modulation of the intracellular antioxidant level did not influence classical NF-κB pathway activation. As ROS are highly reactive and extremely short-lived, stronger proof for this concept might be obtained from future *in vitro *co-culture experiments where macrophages and type II epithelial cells are cultured simultaneously, so that macrophages are allowed to adhere to lung epithelial cells, providing an oxidative stress setting more similar to the *in vivo *situation. In a mixed co-culture of primary rat macrophages and primary epithelial type II cells however, no significant induction of several proinflammatory genes, including iNOS, was found after silica treatment [55]. The discrepancy between these investigations and the current study might be due to the different experimental set-up. Although primary cells are of higher biological relevance, the isolation of these cells might induce stress-related genes in the cells, which could make it harder to observe differences between treated and control cells. Another possibility is that direct contact of macrophages and epithelial cells diminishes iNOS induction by quartz treatment, possibly due to a lower activation of macrophages by surfactant production, which was shown in a follow-up to the former study [56].

In previous studies, we have demonstrated that modification of the surface of DQ12 quartz particles, e.g. using the polymer PVNO, leads to a marked inhibition of inflammation and toxicity in rat lungs after instillation [21,24,57]. In the present study, we found marked effects of particle surface modification in our *in vitro *model: When cells were treated with supernatant from PVNO-quartz-treated macrophages, IκBα phosphorylation and degradation was abolished, compared to the effect of supernatants obtained from macrophages that were treated with the non-coated quartz. Also, the induction of the two main genes induced by macrophage supernatants in our system, iNOS and COX-2, was found to be abrogated. A similar inhibition was found for direct quartz treatment, as HO-1 and COX-2 induction was almost brought back to the control level. Our current findings with the PVNO-modified particles point towards a specific effect of quartz particles, and suggest that particles of lower toxicity or reactivity may not trigger either the macrophage-driven or direct particulate effects on the epithelial cells. Currently we are investigating this by evaluating a set of particle types with different size and surface properties. Altogether our study indicates the existence of a differentially mediated pro-inflammatory activation of lung epithelial cells in quartz-exposed lung, which in both cases appears to be driven by the particle surface properties: Firstly, quartz particles can induce cellular oxidative stress leading to an NF-κB-independent increase in expression of HO-1 and COX-2. We hypothesise that the transcription factor nuclear factor E2-related factor 2 (nrf-2), which was elegantly shown to be the main transcription factor regulating HO-1 expression [58], is at least in part involved in these observed direct quartz effects. Secondly, stable mediators released from quartz-treated macrophages strongly induce the classical NF-κB pathway as well as iNOS. Remarkably, the involvement of the most likely candidates for this indirect, macrophage-mediated activation, i.e. TNFα, IL-1β and H_2_O_2 _could be ruled out. In further experiments we could also exclude potential contributions of macrophage-derived NO as well as of proteases, which both have been shown to be involved in activation of the NF-κB pathway [59,60]. In line with observations by others who have used primary macrophages [61], quartz did not elicit any detectable NO production from quartz-treated NR8383 cells, measured using the Griess assay, in contrast to lipopolysaccharide (LPS, 0.1 or 1 g/l) (data not shown). The effect of proteases could be ruled out by the addition of protease inhibitors to the supernatants of quartz-treated NR8383 cells (data not shown). Among various potential candidates, one could also consider prostaglandins (PG), which are known to be released from macrophages. However, there is considerable experimental data suggesting that many of the arachidonic acid metabolites rather act as NF-κB inhibitors and thereby provide a negative feedback loop for COX-2 expression [62,63]. Indeed, the well investigated metabolite PGE2, which is considered as an acute pro-inflammatory mediator, has been shown to increase the transactivation potential of the RelA subunit of NF-κB in intestinal epithelial cell lines. However, this effect was demonstrated to occur in the absence of IκBα degradation and nuclear accumulation of NF-κB [62]. Thus, macrophage-derived prostaglandins including PGE2, unlikely explain for the observed macrophage-mediated phosphorylation and degradation of IκBα and subsequent translocation of RelA in the RLE cells. Although the responsible mechanism is yet to be identified, our data indicate that for therapeutic or possible preventive approaches, an intervention at the macrophage product level might not be feasible, as targeting of the main individual mediators suspected to be involved (TNFα, IL-1β) was unsuccessful. Additionally, in such intervention direct particle effects on epithelial cells (e.g. COX-2 induction) would persist. Instead, a more promising strategy should focus on upstream processes, e.g. by targeting the initial activation of macrophages by the quartz particles. Preferentially, one should aim at blocking the secretion of mediators from macrophages without interfering with their phagocytic properties that are essential for lung homeostasis and are known to provide the hallmark of particle clearance out of the alveoli via the lymph nodes. Recent *in vitro *investigations in our laboratory [64] indicate that this may possibly be achieved by targeting Fc II-receptor signalling pathway in the macrophages.

## Conclusions

The data presented in the current paper show, on the one hand, that macrophage products secreted in response to quartz treatment induce NF-κB and iNOS in rat alveolar type II epithelial cells, unlike the quartz particles themselves. On the other hand, the particles, rather than macrophage mediators, markedly induce HO-1 and COX-2 mRNA expression in epithelial cells in the absence of NF-κB pathway activation. Taken together, our results suggest that although both oxidative stress and NF-κB are likely involved in the inflammatory effects of toxic respirable particles, these phenomena can operate independently on the cellular level. This indicates that *in vitro *testing strategies to predict the inflammogenic potency of particles should not exclusively target the NF-κB pathway. Our findings also underscore the importance of further elucidation on the involvement of NF-κB-independent signalling pathways in the development and progression of pulmonary disorders induced by toxic particles such as quartz.

## Abbreviations

DQ12: Dörentrup Quartz "ground product nr. 12"; Q: quartz particles, see above; PVNO: Polyvinylpyridine N-oxide; Q-MS: supernatant of quartz-treated macrophages; c-MS: supernatant of non-treated control macrophages; ROS: Reactive Oxygen Species; TNFα: Tumour Necrosis Factor alpha; IL-1β: Interleukin-1 beta; HO-1: Heme Oxygenase-1; iNOS: inducible Nitric Oxide Synthase; COX-2: Cyclooxygenase-2; COPD: Chronic Obstructive Pulmonary Disease; AM: Alveolar Macrophage; RNS: Reactive Nitrogen Species; NF-κB: Nuclear Factor kappa-B; IκBα: Inhibitor of κB alpha; BAL: Bronchoalveolar Lavage; BSO: Buthionine Sulfoximine; NAC: N-acetyl cysteine; DMSO: dimethylsulfoxide; HBSS: Hanks' Buffered Salt Solution; DMPO: 5,5-Dimethyl-1-pyrroline N-oxide; DMPO-OH: 5,5-Dimethyl-1-pyrroline N-oxide hydroxide.

## Competing interests

The authors declare that they have no competing interests.

## Authors' contributions

DvB performed most of the experimental work and drafted the manuscript. AK participated in the RT-PCR work. FvS was involved in the study coordination and the RT-PCR analyses. RS was involved in the study design and coordination and helped drafting the manuscript. CA was involved in the study design and coordination, performed the animal experimental work, assisted in the immunocyto- and -histochemistry and helped drafting the manuscript. All authors read, commented on and approved the manuscript.
